# COVID-19 and School: To Open or Not to Open, That Is the Question. The First Review on Current Knowledge

**DOI:** 10.3390/pediatric13020035

**Published:** 2021-06-01

**Authors:** Francesco Busa, Flaminia Bardanzellu, Maria Cristina Pintus, Vassilios Fanos, Maria Antonietta Marcialis

**Affiliations:** Neonatal Intensive Care Unit, Department of Surgical Sciences, AOU and University of Cagliari, SS 554 km 4,500, 09042 Monserrato, Italy; f.busa93@gmail.com (F.B.); cristina.pintus@yahoo.it (M.C.P.); vafanos@tin.it (V.F.); ma.marcialis@libero.it (M.A.M.)

**Keywords:** SARS-CoV-2, COVID-19, children, adolescents, secondary transmission, molecular swab, non-therapeutic intervention, influenza virus, variants, recommendations, psychological aspects

## Abstract

The COVID-19 pandemic has led to an unprecedented closure of schools in terms of duration. The option of school closure, SARS-CoV-2 initially being poorly known, was influenced by the epidemiological aspects of the influenza virus. However, school closure is still under debate and seems unsupported by sure evidence of efficacy in the COVID-19 era. The aim of our narrative review is to discuss the available literature on SARS-CoV-2 spread among children and adolescents, in the school setting, trying to explain why children appear less susceptible to severe disease and less involved in viral spreading. We also tried to define the efficacy of school closure, through an overview of the effects of the choices made by the various countries, trying to identify which preventive measures could be effective for a safe reopening. Finally, we focused on the psychological aspects of such a prolonged closure for children and adolescents. SARS-CoV-2, children, COVID-19, influenza, and school were used as key words in our literature research, updated to 29 March 2021. To our knowledge, this is the first review summarizing the whole current knowledge on SARS-CoV-2 spreading among children and adolescents in the school setting, providing a worldwide overview in such a pandemic context.

## 1. Introduction

In March 2020, due to the SARS-CoV-2 spread, most countries decided to close schools, including Italy. Other nations, instead, decided to adopt targeted closures based on the presence of outbreaks or index cases [1]. The option of school closure was strongly influenced by the analogies existing between COVID-19 and the influenza virus. In the past, school closure effectively contributed to mitigate the diffusion of influenza epidemics [2]. However, the debate is still open and school closure seems not supported by sure evidence of efficacy against SARS-CoV-2.

Although the evident and unquestionable beneficial impact of in-person attendance of teaching programs, for students, the current pandemic forced a thorough risk assessment to avoid the possible outbreak of infections in the school setting. Italian schools of all levels were closed on 4 March 2020 following the Prime Minister’s decree, and the next school year was scheduled to start on 14 and 24 September 2020. To date, in Italy, 73% of youth and children attend school lessons by accessing the remote teaching system (distance learning). The new Prime Minister’s decree, effective on 6 March 2021, established the school closure in those regions considered at high risk of viral spread, leaving the decision to the regional governors in case of mild or low risk regions [3]. In Italy, the school-attending population is included between 3 and 18 years, accounting for a total number of about 8,900,000 subjects [4]. The schools were closed due to the unfounded conviction that children could represent the major vectors of the SARS-CoV-2 domestic transmission, as observed for the influenza virus [5].

Currently, some early Chinese data seemed to confirm the role of children in the transmission of the infection, although they usually present a mild or completely asymptomatic form of the disease [6]. Public opinion and political communication have contributed to keeping schools closed by claiming that schools could act as infection amplifiers. The closure concerned up to 90% of students worldwide, with the exception of Sweden, where such restrictive lockdown measures were not adopted [1].

Observational studies suggest that school closure might achieve a reduction in the incidence of viral transmission. Conversely, studies sustaining school reopening highlight that the disease is generally mild in children, emphasizing that children play a less important role in the infection’s transmission, if compared to adults. Even transmission from children to teacher or other members of the school staff would not seem statistically significant [7].

Following the first wave of SARS-CoV-2 diffusion, the choice to reopen schools differed from country to country, as did the choice to apply dedicated medical procedures. The schools were closed once again after the identification the UK viral variant, despite lack of information concerning the exact impact of the B.1.1.7 variant on children [8].

In this narrative review, we aimed to discuss the available literature on SARS-CoV-2 spread among children and adolescents, and especially in the school setting. We investigated why children seem less susceptible to severe disease and less involved in secondary transmission; we also tried to define the efficacy of school closure, through an overview of the effects deriving from the different choices adopted by the various European and extra-European countries, trying to identify which, among the preventive measures recommended, could be effective for a safe school reopening, also taking into account the effects of the vaccinations programs and the new emerging viral variants. Finally, we also focused on the psychological consequences of such a prolonged school closure in children, adolescents and their families.

Moreover, it appears clear that online learning favours urban and more developed areas, due to a lack of network’s resources, the unavailability of electricity and electronic devices in rural areas, widening the disparity between the poor and the rich, instead of uniting the nation in the fight against COVID-19 [9]. Especially the rural students who do have a family schooling background are at a greatly disadvantaged position. For example, the Ethiopian government failed to make interventions to support the marginalized rural students of any level of education [10]. Ethiopia also recommended private schools to explore methods to either cancel fees or defer payments until parents can afford to pay. Indeed, reduction of incomes due to the COVID-19 economic crisis may also lead to shifts in enrolment from private schools to public schools, adding further pressure on public education finances [11].

In Table 1, we summarized the available studies investigating SARS-CoV-2 transmission in the school setting. SARS-CoV-2, children, COVID-19, influenza, and school were used as key words in our literature research, updated to 29 March 2021.

## 2. Children Susceptibility to SARS-CoV-2

The precise role of children in the transmission of SARS-CoV-2 is not fully known, but they have a lower susceptibility in comparison with adults, and they seem less able to transmit the virus [25].

In the analysis of 72,314 COVID-19 cases in China, children were found as a minority of the confirmed cases; in fact, subjects between 0 and 19 years old were between 0.9% and 1.2% of the total number of cases [6]. Similar results were later confirmed by other authors [26,27].

Davies et al. estimated that susceptibility to SARS-CoV-2 infection in people younger than 20 years of age is 50% lower than in adults [28], but it must be considered that it is a wide age range, not considered only children. A similar finding was also confirmed by Dattner et al. through a stochastic dynamic mathematical model of a family infection outbreak, finding reduced susceptibility and infectivity in comparison with adults, by 43% and 63%, respectively [29]. The lower infection rate in children was also highlighted by a wide serological investigation carried out on 61,000 Spanish subjects [30], in which antibody positivity in children and adolescents was 3.4%, versus 4.4–6.0% in adults.

In a “picture” of the SARS-CoV-2 epidemic in Italy [31], until 8 May 2020, paediatric subjects under 18 years (3836) accounted for 1.8% of all positive cases in the population (216,305), with a median age of 11 years. To date, the scenario results completely changed, with a percentage of 16.8% in the 0–18 age group, 44.2% in the 19–50 age group and a rate of 39.0% in the age group over 50 years [32].

The analysis performed by the Italian Superior Health Institute (ISS), aiming to describe the infection distribution among subjects under 18 years, reported that teenagers between 13 and 17 years had a positivity rate of 41.3%, followed by children aged 7–12, with a rate of 28.0%, children aged 2–6 with a rate of 21.0% and children aged 0–1 with a positivity rate of 6.7% [33].

A literature analysis showed that a significant proportion of SARS-CoV-2 positive children do not develop symptoms. Most of them show a mild or moderate disease, whereas critical forms are very rare, just like deaths [34].

A very recent meta-analysis discussing seventy-one studies, including 11,671 paediatrics subjects, showed the incidence of symptoms among children and adolescents: respiratory symptoms were reported in 56.8% of cases, fever in 55.8%, gastrointestinal symptoms in 14.4%, symptoms attributable to the nervous system in 6.7%, thoracic constriction in 6.1%, whereas asymptomatic patients were 21.1%. In this meta-analysis, the rate of multisystem inflammatory syndrome in children (MIS-C) was also very relevant, and more frequent than previously described, occurring in about 6.2% of cases [34].

On the contrary, according to another recent review, a severe disease tends to develop in a lower percentage of subjects, representing about 1% of cases and, apparently, the severity of the infection in the various age groups follows a “U-shaped curve”, with newborns and infants being more affected and more at risk of hospitalization [25]. Finally, some anomalous laboratory values were reported, like leukopenia and lymphocytopenia (8.8% and 12%, respectively) [34,35]. Among children, infants seem more vulnerable and more at risk of severe forms, with a higher rate of hospitalization if compared with older ones [36]. A recent analysis of a paediatric population of 50,000 subjects under 18 years, in the city of Los Angeles [37], showed a percentage of 54.6% totally asymptomatic children. In a review [38], 31 studies conducted in 21 different countries were discussed, showing a lower susceptibility rate to SARS-CoV-2 infection among children, with an odds ratio (OR) of 0.56 (95% CI, 0.37–0.85), identifying subjects younger than 10–14 years old as the category with minor absolute risk. Adolescents, instead, seem to be closer to the infection susceptibility curve of adults.

In a meta-analysis by Thompson et al. [39], by examining the diffusion of COVID-19 within families, some further evidence of a dependency between age and susceptibility to infection was found. However, there is still low confidence about this data; therefore, understanding the mechanisms of the infection with reference to the paediatric age is an important challenge to face in the next few months.

### 2.1. Children-Related SARS-CoV-2 Transmissibility

Unlike previous hypothesis, in addition to the frequency and severity of the disease, the SARS-CoV-2 spreading speed is also lower in children than in adults.

Multiple studies proved a correlation between old age and an increase in the viral RNA load, by sampling the nasopharyngeal tract and the respiratory tree [40,41,42,43]. Subsequently, a systematic review [44] hypothesized a correlation between average age and average duration of the spread of the SARS-CoV-2 RNA from the upper respiratory tract, thus suggesting less propagation from children than from adults. On the other hand, children may have a prolonged virus spread via the gastrointestinal route [45].

In February 2020, a China/World Health Organization (WHO) joint commission was not able to report any cases of child-to-adult transmission [46]. Subsequently, Danis et al. reported the case of a SARS-CoV-2-positive 9-year-old child with a picornavirus co-infection and type A influenza, affected by mild symptoms, who did not transmit the infection to any of his 172 close contacts (including two siblings). While he was symptomatic, the child attended three different skiing schools, and all contacts were quarantined as a precaution, without developing the disease [47]. A South Korean study found only one positive case (regarding an adult patient) among the 58 tight contacts of a SARS-CoV-2 positive 9-year-old girl [48]; similarly, a Thai study involving three patients hospitalized due to mild to moderate symptoms, investigated the possible infection of health operators without detecting any positive cases [49]. In agreement with these reports, a large contact-tracing Indian study concluded that the highest probability of SARS-CoV-2 transmission is found among people of similar age, especially in the over 65 and in the 0–4 age groups [50].

The transmission from a child as an index case within the family has been scarcely investigated and, in any case, few clusters of families infected by their children have been found, as reported in studies conducted in the USA [51], Israel [52] and Greece [53]. In this context, a South Korean study involving 11,000 participants found a percentage of transmission of 11.8% among family contacts; among these, in only 5.3% the index case was represented by a child aged < 9 years; if the child’s age was between 10 and 19 years, the transmission rate was found to be equal to 18.6%, the highest in the study [54]. Finally, a large study carried out in Germany suggests that transmission within the family is scarcely frequent. The study, involving 15,771 children (<18 years), found a transmission rate of 35% (assessed by determining anti-SARS-CoV-2 antibodies) in family members living with SARS-CoV-2-positive paediatric subjects [55].

As a result, even if the information about transmission of SARS-CoV-2 from a paediatric index case is heterogeneous, children do not seem to be particularly contagious.

### 2.2. Host and Guest: Why Are Children Less Affected and How Long Do They Host SARS-CoV-2?

Undoubtedly, children have a lower comorbidity rate, as well as a healthier respiratory system with a great recovery and tissue repair potential following a viral infection, which has also been less exposed to cigarette smoke and pollution. The thymus gland, where T cells develop, has a higher absolute weight in children aged 6 to 13 years and the preservation of a good T cell function might explain why children are less susceptible to SARS-CoV-2 infection [56]. Their greater immune response against protein vaccines, like the anti-papilloma virus and the anti-hepatitis virus, indicates that early immunization may result in permanent protection. Moreover, children have a “well-trained” immunity, both due to their more frequent exposure to pathogens in kindergartens and schools, and due to their repeated vaccinations [57].

Gold et al. introduced the theory that the trivalent measles–mumps–rubella vaccine might be associated with a reduction of deaths due to COVID-19 [57,58]. The same author confirmed, in a later study, the presence of an unexpected protection against SARS-CoV-2 due to the anti-mumps antibodies, with a significant inverse correlation between antibody titre and severity of COVID-19 [59].

Typically, the immunity due to childhood vaccines lowers as the age increases, therefore the risk of COVID-19 in elderly people increases. The immune system also seems to undergo an immunosenescence process which makes it less dynamic in producing antibodies and/or virus-specific T cells (CD8+), which play an important role in the viral clearance, by directly eliminating infected cells. In children, a relative preponderance of T CD4+ cells, reduced infiltration of neutrophils, reduced production of pro-inflammatory cytokines and an increase of immunomodulatory cytokines are observed. Cytokine levels, including IL-4, IL-6 and the Tumour Necrosis Factor alpha (TNF-α) are rarely high [60,61].

In children, the intestinal microbiota seems to play a protective role against SARS-CoV-2. In fact, it differs from that of elderly people, which is defined as “fragile” because it has been impoverished, especially in terms of biodiversity, and may also be modified by several comorbidities (diabetes, hypertension and obesity), which often lead to gut dysbiosis. Recent works underline that the intestinal microbiota can interact with the viruses that reach the intestine, affecting their virulence and invasiveness [15,62]. Other studies have reported significant alterations of the faecal microbiota in patients with COVID-19 during hospitalization, which vary according to the disease severity [63]. In conclusion, we cannot exclude that children’s intestinal microbiota may play a protective role against SARS-CoV-2 and, probably, we could consider it as an unrecognized player [16].

### 2.3. Methodological Limitations

The large amount of data proving asymptomatic or mildly symptomatic SARS-CoV-2 in children is highlighted by the epidemiological data reported above; however, the low infection incidence among paediatric patients might be due to the low number of swabs performed in this population, due to the general policy to test symptomatic cases exclusively.

In an investigation by Li et al., some fully asymptomatic students and staff members were randomly tested in 100 English schools, finding a positivity rate of 1.24% and 1.29%, respectively, similar to the 1.2% positivity rate found in the general population [64]; this finding detected a greater susceptibility in paediatric patients than commonly reported.

At the same time, the contacts in the school setting do not seem to represent significant risk factors for the onset of new epidemic outbreaks. Moreover, the swabs based on RT-PCR tests among children entail a non-negligible number of false negatives, unless the tests are repeated. Such a under-detection might also be due to the fact that children have milder symptoms and remain more isolated during school closure. Some authors recommend a combined evaluation of RT-PCR and antibody title and suggest that a lack of detection of both diagnostic tests, in particular in children under 6 years, might entail errors [29]. An alternative approach to examine susceptibility to the infection is to use contact tracing studies, in which the contacts of known cases are isolated and tested.

## 3. Arguments in Favour of School Opening. What Does It Depend On?

Not all countries worldwide applied the same restrictive measures to limit infections in the paediatric population, even though most of them decided to close all types and all levels of schools.

In this section, we discuss the international literature sustaining school reopening during the pandemic, starting from Sweden, which preferred to keep children and teenagers in schools (kindergartens, primary schools and first few years of secondary schools), considering the growing evidence of mild infections in paediatric age and the potential negative psychological consequences due to the closure, especially in younger children. Moreover, though social distancing was encouraged, wearing masks was not mandatory in Swedish schools [65]. To date, the impact of such a strategy is not fully known, but the pandemic does not appear to have a worse outcome in Sweden in comparison with the countries in which more rigid measures have been introduced. Through the data published by the Swedish Health Agency, the study of Ludgviggson et al. compared the number of hospitalizations and mortality rate in the 0–16 years age group between March and June 2020 and in the four months before the COVID-19 pandemic (November 2019–February 2020), finding comparable numbers and not detecting any deaths attributable to COVID-19 among children [1]. A report by the same Swedish Health Agency, not peer-reviewed, found 1124 cases of SARS-CoV-2 in children and teenagers aged 0–19, or 0.05%, in the period between 24 February and 14 June 2020, that is the same infection rate as Finland, and reaching the conclusion that closing schools seems to not affect viral spread [66].

In Europe, even after the second viral wave, various countries have taken different decisions about reopening schools. Denmark was the first state to let kindergarten and primary school children go back to school, as early as on 15 April 2020. Norway and France decided to keep their schools open most of the time during this year of pandemic (only closing them for 35 and 50 days, respectively), whereas Germany and the United Kingdom ordered schools to be closed for 105 days [67].

In Norway, from 20 August to 20 November, a period with a low overall SARS-CoV-2 incidence and in which it was decided that children with symptoms should stay at home, a 0.9% infection rate was found in 234 paediatric subjects taken into consideration in the primary schools of Oslo and Viken [20]. Moreover, in November 2020, 11% of schools within the national territory reported at least one positive case among children and staff members, but less than 1% of these reported clusters with more than 9 cases [21]. After the epidemiological peak of infection registered during the second European wave, which took place in the first week of November 2020, the incidence rate among children and adolescents fell by 60% in the following 4 weeks, a period in which all schools in the country were open. On the contrary, a 125% increase of infection rate was detected in the week of Christmas and the first week of the year. This is a period of school holidays, with an increase of social contacts among adolescents outside school. Finally, the exact determination of the setting of infection, whether transmission occurred in the school setting or in the community (or family) could be precisely determined by a genotype evaluation of the virus isolated from a nasopharyngeal swab of positive subjects [68].

In Germany, between May and June 2020, the sera of 1530 students and 507 teachers were collected, to assess the anti-SARS-CoV-2 IgG antibody response, finding a positivity rate of just 0.6%, thus concluding that schools play no decisive role on pandemic trends [69]. Similarly, until May 2020, in the Baden-Württemberg province (Germany), 453 children were found positive for SARS-CoV-2 and attended their respective schools for at least 1 day during the infective period. Over 2300 nasopharyngeal swabs were thus collected from the close contacts of 137 index cases. Among these, six subjects infected only 11 children (0.5%), reinforcing the concept that that child-to-child transmission in school is rare and is not the first cause of infection among paediatric subjects [17]. Seroprevalence studies (which enrolled about 5000 subjects) estimated a prevalence of anti-SARS-CoV-2 antibodies in the parents of children attending school in Germany equal to 1.8% and a percentage three times lower in children, that is 0.6% [18].

In Ireland, in order to ascertain whether infections were spreading in schools, the six SARS-CoV-2 cases notified to the Public Health Agency relative to subjects who attended school before closure were taken into consideration. In particular, the three pediatric cases and the three adult staff members came into contact with another 924 children and 101 adults during classrooms lessons and physical education lessons. Among these 1001 close contacts, no cases of COVID-19 were found [70].

In Madrid, Spain, in addition to the abovementioned investigation on the antibody positivity in the first 2 weeks of March 2020, a study showed that only 41 cases out of 4695 confirmed ones (positive test) were younger than 18, or 0.8% [71].

A very recent Italian study, performed from 30 September 2020 to 28 February 2021, involved two provinces (Trento and Bolzano) with similar climate, population density and lifestyle, but in which schools reopened at different times. In fact, in Trento, they reopened one week later than Bolzano. This study did not find any unquestionable and constant time relationship between the opening of the schools and an increase in the transmission rate (RT) index in the general population. Later, this finding was also confirmed by an extension of the study to wider areas of Italy. Similarly, closing schools in some Italian regions like Campania and part of Lombardy did not have any effect on the general population infection rate or RT index, which increased before the closure and continued to grow despite the implemented measures [19].

In Wisconsin (USA) from 31 August 2020 to 29 November 2020, 17 schools were studied, in which strict protocols were applied making surgical masks mandatory. A decreased number of infections, in comparison with the general data for the whole county (3453 cases vs. 5466 cases per 100,000 inhabitants), was detected, and the 191 identified index cases had transmitted the infection to only seven more subjects; therefore, the secondary infection rate was very low (3.7%) [72]. These encouraging findings were also confirmed in a study involving over 90,000 students in North Carolina, where after the first 9 weeks of “physical” school attendance, only 1% of SARS-CoV-2 positive cases and 4.1% of secondary transmission were detected [73].

Another American study proved that, until 29 October 2020, the group of people entailing an upsurge of the pandemic, which resulted in reproduction numbers constantly over 1, was composed by adults aged 20 to 49 (especially 35 to 49). Up to 72.2% of infections were attributed to this group of patients, whereas less than 5% were caused by children aged 0 to 9 and less than 10% by teenagers between 10 and 19, thus leading to the conclusion that schools seem not to cause an increase in the infection curve [74].

In a case–control study carried out in Mississippi [75] involving 397 children and adolescents in the September–November 2020 time period, it was noticed that in the “case” group (subjects positive to SARS-CoV-2), there was a higher probability than in the “control” group (negative subjects) to have received visitors at home from outside (OR 1.95), to have performed entertainment activities with other children (OR 3.3) and social activities (OR 2.4); all these activities occurred outside of school. In particular, the conclusion of this study highlights that school attendance in kindergarten, during the 2 weeks before a SARS-CoV-2 positivity, was not associated with an increased infection spread.

The latest data provided by the Australian government, in a note on its official site, report a COVID-19 incidence rate among school children (aged 5–17) of 4.5% of total cases [23]. In Australia, despite the lower students’ attendance to school during the peak, most schools remained open during the first pandemic wave. During this phase, in the Australian state of New South Wales (8.1 million inhabitants) a study was carried out on students and staff members in 15 schools and 10 early childhood education and care (ECEC) structures, finding positive swabs for 12 children and 15 adult staff members while they were considered infective (precisely 24 h before the onset of symptoms); subsequently, their 1448 close contacts were monitored: among these, 663 (43.7%) were tested, with 18 ascertained cases of SARS-CoV-2 (1.2%) transmission, thus proving a low secondary transmission risk [76].

## 4. Arguments in Favour of School Closure. What Does It Depend On?

Despite the uncertainty about the efficacy of school closure, in March 2020, schools were closed in all the 50 states of the USA. In an observational study carried out from 9 March to 7 May 2020, closures were found to be temporarily associated with a reduction in the incidence rate (adjusted relative change per week, −62%) and the mortality rate (adjusted relative change per week, −58%) due to SARS-CoV-2 infection [24].

Gurdasani et al. were doubtful about the complete reopening of schools, announced by the British government on 8 March 2021. Some political movements and the public opinion exerted some pressure to preserve school education, which, however, also resulted in some less strict preventive measures in schools. However, primary and secondary schools closure has always been associated with a decreased RT index in most countries (including England). Before Christmas 2020, the British Office for National Statistics noted an increased prevalence rate in the 2–10 years age group (2%) and in the 11–16 years age group (3%), which exceeded that of the general population [22].

In the USA, by 4 February 2021, the COVID-19 positive children were almost 3 million and accounted for 12.9% of all infections taking place in the United States. A rapid growth of cases, of about 10%, was noticed in the last week of January 2021 and the first week of February [77].

A wave of infections was also noticed in Israel 10 days after schools reopening, in May 2020. In Jerusalem, schools were reopened on 17 May 2020, and a measurement of infections before 24 May 2020 showed that positive cases among people aged 10 to 19 were 19.8%, while the incidence rate had grown to 40.9% in the following 3 weeks [12]. In addition, a warning was recently published, on 9 February 2021, to draw attention to the reopening of schools in Israel and Italy, since these countries had a higher percentage of young people infected with the new COVID-19 variants (e.g., In Israel, in mid-December, the proportion of new daily cases accounted for by children aged under 10 had risen by 23%) [13].

The need to close schools may be also supported by a Chilean work published by Oxford Academy [14], which analysed the outbreak affecting 52 people in a school community in Santiago. The school was closed on 13 March 2020 and all the students were quarantined and then underwent an antibody test for IgM and IgG. The antibody positivity was 9.9% in the 1099 students and 16.6% in the 235 staff members. The higher incidence rate in the latter group seems to be attributable to the fact that the index case was a teacher.

Finally, a study by Ladhani et al. [78] actually highlighted an equal infection rate in adults and children (by similar antibody positivity figures), although the latter develop the disease in an asymptomatic or paucisymptomatic way in half of the cases.

## 5. Taking Attendance: SARS-CoV-2 Present or Absent at School?

Almost all the studies currently available in literature and dealing with SARS-CoV-2 school spreading are descriptive/observational studies. The authors followed infection trends in the various European and extra-European countries among students, teachers and school staff members after reopening schools for a variable time period and detected the SARS-CoV-2 incidence rates. No experimental studies were found in which investigators played an active role by introducing new prevention measures or excluding some of them, mostly due to ethics reasons. What is important to underline is that the presence of multiple COVID-19 cases in a school setting does not necessarily mean that the transmission occurred in school; research studies on the topic aim to reconstruct the direction of the infection and the setting where it occurred.

Starting on 5 October 2020, in the first month after schools reopening, 1212 children out of 65,104 in Italian schools were found to be SARS-CoV-2 positive (1.8%). The distribution of these cases at the various school levels was the following: 17.5% in kindergartens, 22.2% in primary schools, 15.4% in middle schools and 33.5% in secondary schools, 4.1% peer institutions, 7.3% not available [79]. In the same month, taking into consideration all Italian schools, in over 90% of cases only one isolated SARS-CoV-2 infection episode was reported in individual schools; only in one secondary school was a cluster of more than 10 positive subjects described [80]. Therefore, in our opinion, the data collected in this review suggests that, when preventive measures are correctly applied, transmission within a school class is quite low, and also strengthens the previous hypothesis that children are less susceptible to the infection.

A study by Villani et al. on two schools in Rome processed 3431 swabs collected in 3 months (21 September–4 December 2020), detecting a total of 16 positive samples; all these children attended different classes. According to the authors, these observations suggest that schools do not amplify the infection when preventive measures are applied [81].

An Italian survey conducted between 14 September and 15 October 2020 analysed 41 classes of 36 schools in the province of Reggio-Emilia [82]. It is interesting to underline that, among the study methods, the following criteria were applied: when a positive nasopharyngeal swab was detected among students or staff member, all classmates and close contacts of the index case at school were immediately tested and then retested 14 days later. Starting from the findings of the various index cases, 994 swabs were collected from children, finding 38 positive cases among them (3.8%), whereas no secondary transmission took place among teachers. As regards the overall incidence rate, it was found to be 6.6% in secondary schools and 0.38% in primary schools. It was then concluded by the authors that a non-negligible number of infections with SARS-CoV-2 took place in schools, especially thanks to the implementation of uniform diagnostic protocols.

This uncertain opinion about reopening schools in Italy is put forward in a work by Sebastioni et al. [83], in which there is a strong belief that schools are the main factor triggering SARS-CoV-2 spread, especially due to the use of public transport by students. To support this hypothesis, the 2 weeks period between school reopening and the start of an exponential growth of infections was considered, representing a comparable time window between the first national lockdown in March 2020 and the peak incidence rate in the first wave. The reopening of schools was considered to be the only “new fact” which took place in the country sufficiently significant to affect the infection curve.

The analysis performed in the Piedmont region, by a researcher of the University of Turin [84], is also remarkable. He reported that the incidence rate among secondary school students almost doubled in comparison with that of the general population of the same region, starting from the week of 12 October 2020. On 16 October, the Piedmont region, not being able to keep up with the swab processing anymore, modified the provisions and decided to re-admit to class those students who had completed the quarantine, even without a control swab. Due to this reason, in the following week, the incidence rate fell (probably also due to the lower number of processed swabs), but was still higher than that of the general population. Only in the next few days, when distance learning was introduced, did the school infection curve fall below that of the general population. Despite this observation, we can state that, in such a situation, it is difficult to highlight a strong correlation between the variables in question: infections, disease and social interactions. However, in Italy, detecting one or more positive cases at school, along with the epidemiological data in the national territory, led to the closure of many schools. This is in contrast with what is envisaged in the guidelines of the European Centre for Disease Prevention and Control (ECDC), which only recommend isolation of all asymptomatic or suspected positive close contacts for 14 days [85].

### COVID-19 at School: Who Infected Who?

In Italian schools, during the second wave of the pandemic, infections were found to be more widespread among teachers and staff members than among students. In particular, compared to the general population it was found out that elementary and middle school students had a 39% lower infection rate and the secondary school students had 9% lower [19]. An Italian study carried out during the second pandemic wave clarifies that, in the Veneto region, children and adolescents were not early carriers of the disease. On the contrary, the infection was associated with a high incidence rate in the 20–29 and 45–49 years age groups and, in the case of teachers who had been infected at school, the authors concluded that the infection came from other teachers rather than students [19].

In an English study, performed after the schools reopened (from 1 June to 17 July 2020), 113 cases of SARS-CoV-2 infections were detected in 38,000 kindergartens, 15,600 primary schools and 4000 secondary schools students, with about 928,000 students daily attending them, and school staff members had a higher incidence rate of the disease in comparison with students (8.4% primary and secondary staff school members versus 3.1% in primary school students and 3.9% in secondary school students). Most cases were outbreaks starting from staff members and spreading among staff members themselves; among these outbreaks, transmission occurred from students to staff member in 16 cases and from student to student only in five cases [7].

An investigation carried out in Georgia, USA [57] monitoring 2600 students and 700 staff members for a period of 2 months while schools were open, showed that the COVID-19 incidence rate in the general population of Cobb County increased by 300%. In particular, nine clusters were identified, which involved 13 educators and 32 students in six schools out of the eight investigated schools; in four of these clusters, the index case was found to be an educator, suggesting that they play a key role in viral spreading when compliance with preventive measures is not impeccable.

A recent Scottish study estimates the risks of hospitalization or severe COVID-19 in teachers compared to healthcare workers or to the general population. Most teachers were young (mean age 42), female and had no underlying conditions, and it was found that they are not at increased risk of hospitalization with COVID-19 (rate ratio, RR 0.97) and are at lower risk of severe COVID-19 (rate ratio, RR 0.27) [86].

## 6. The Influenza Virus and Decision-Making during the COVID-19 Pandemic

Due to the rapid spread of the SARS-CoV-2 virus, most governments worldwide took the decision to close all school levels. This decision was also based on the pandemic spread model of the Influenza A and B virus, where children seem to be most frequently affected and responsible for transmission in the majority of cases [2], even though the issue is still under debate [87].

To date, even though the first epidemiology data on the general population have shown more serious clinical indices for SARS-CoV-2 compared to the influenza virus [88], there are still very few certainties about its real impact on paediatric age, and divergent data on COVID-19 do not allow for a comprehensive understanding of the issue. Unfortunately, regarding influenza, paediatric population data are hard to interpret [89], although its high incidence on this group is extremely clear. A three-year prospective study carried out in Hong Kong estimated that the cumulative infection incidence in the subjects enrolled (aged between 5 and 17) was 59% during the first wave of H1N1 in 2009, then 7%, 14%, 20%, and 31% during the subsequent epidemics of H3N2 (2010), H1N1pdm09 (2011), B (2012) and H3N2 (2012), respectively [5]. Following the same approach, a study on the varying incidence of the influenza virus among children (203 subjects enrolled, aged between 5 and 17) and adults (413 subjects enrolled, aged between 18 and 59), based on antibody testing, showed an overall incidence rate of seasonal influenza of 31% and 21% in the two groups [90]. Several prototypes have been designed to describe how school closure might lead to a reduction in the incidence peak of the influenza virus pandemic; an example of this is the study by Ferguson et al. [91], pointing to a drop by as much as 40%.

Epidemiology data that supports this approach are also available; for example, an Israeli study lasting two weeks, where the closure of schools involved children aged between 6 and 12 and determined a reduction by 50% in the diagnosis of viral infections and by 28% in visits to paediatricians [92].

Regarding COVID-19, since the latest update by the Royal College of Paediatrics and Child Health (RCPCH) on 12 March 2021, no substantial changes occurred in the global incidence of the virus among the paediatric population, with a value ranging between 1 and 5% [93]. The study published on Nature at the end of June 2020 [28], based on data from six countries (Italy, China, Japan, Singapore, South-Korea and Canada), also showed that subjects under 20 years of age appeared to be half as susceptible to the SARS-CoV-2 infection compared to other age groups. This study also included a simulation of the effect of school closure, for three months, on the spread of both viruses. In the case of the influenza pandemic, the incidence peak fell by 17–35% and was delayed by 10–89 days; regarding COVID-19, the incidence peak reduction was lower—10–19%—and delayed by 1–6 days, which supports that the school closure actually has only a secondary effect on the infection management.

## 7. SARS-CoV-2 Management at School

The school management of SARS-CoV-2, to avoid its spread, is composed of two steps. The first involves handling a “suspicious” case (a child showing typical symptoms of COVID-19). In this case, an isolation room is necessary to isolate the subjects until the swab results are ready [94]. Moreover, in all countries, experts agree that children should refrain from attending classes if they show symptoms.

The second step involves handling a “confirmed” positive case; in this case, the time required before readmission to school varies among the various countries. More specifically, such a period ranges from 7 days in France [95], to at least 10 days in Spain [96], and 14 days in countries like India [97].

All countries require quarantine for close contacts of the index-case, except for Luxembourg. In this country, none of the contacts are isolated; however, the whole class (staff and students) is tested with a molecular swab; if the latter proves negative, classes may resume [98]. This approach opens the debate about whether the decision to have preventive quarantine, as happens in the other countries, may indeed be based on economic considerations.

### 7.1. Fresh Air Could Be the Key to Reopen Schools Safely?

One of the essential recommendations by all European countries is to focus on the optimal ventilation of school premises. Keeping doors and windows open in the classrooms allows cutting down by half the total droplets in approximately 30 s; on the contrary, in a not well-aired environment, particles have a half-life of 4 min [99]. This may be impossible in wintertime, when it gets too cold outside [100]. One interesting approach could be based on the filtration systems on aeroplanes, where the risk of COVID-19 infection is markedly lower than in buildings such as offices or schools [101]. The International Air Transport Association (IATA) decided to introduce, in addition to conventional prevention measures such as a negative test before boarding, use of face covers during the flight and careful hand hygiene, the most important novelty in terms of appropriate airing, namely fresh air recirculation and recycling in the form of High Efficiency Particulate Air [102]. Handheld HEPA filtering units appear to be an ideal solution for air filtering, placed depending on the size of the room, number and age of the people inside it [103].

### 7.2. Other Recommendations to Contain SARS-CoV-2 Transmission in Schools

In Italy, the first consensus document on the prevention and protection of SARS-CoV-2 spread after school reopening was drafted by technical-scientific committee (CTS) on 28 May 2020, in which the importance of physical distancing by at least one 1 m between students and between staff members was highlighted. The importance of face covers, especially when moving around the school (when physical distancing is impossible), was underlined and, in this regard, “students were always expected to wear a face mask in school, except during physical education classes and while on a lunch break” [104]. Nevertheless, in a review by the World Health Organization (WHO) dating back to 21 August 2020 [105], the following guidelines were provided regarding the use of masks, depending on age: children up to 5 years of age are not required to use a mask; among children between 6 and 11 years of age, face mask use depends on the local epidemiology situation, paying specific attention to its impact on a child’s learning ability; finally, children of 12 years old and more have to follow the same measures for adults.

These recommendations by the WHO, together with the United Nations Children’s Fund (UNICEF), are becoming widespread in several countries in Europe and other continents, which recently updated their instructions regarding the use of face masks [105]. Another important measure for prevention is hand hygiene at regular intervals, which is something children should be advised and encouraged to do, especially in younger age groups where contacts with the surrounding environment are multi-faceted and heterogeneous [97].

## 8. A Changing Picture: COVID-19 Variants among Children

The impact of COVID-19 variants in a school environment is uncertain. Although the media have reported an increase in hospital admissions and a rise in serious forms of respiratory disease, it is not yet clear if there might be a correlation between the viral spread and the school attendance [106].

According to a survey by Davies et al., the new variant (“traditional UK variant”, VOC 202012/01 also called B.1.1.7), firstly recorded in the south of England, appears to be 56% more transmissible than the pre-existing variants. Its involvement in a more or less serious form of the disease is not yet clarified; nevertheless, the authors have concluded that its higher transmission rate may lead to the failure of national control measures, unless vaccines start to be inoculated and closure of primary, secondary schools and universities is implemented [8]. According to a report by the Public Health England, the transmissibility of this viral variant, in subjects older than 20 years, is 39% higher compared to the wild-type SARS-CoV-2; on the other hand, among children of school age younger than 10 years, it appears to be 46% lower than among adults [107].

More recently, data from the United Kingdom seem to indicate that children are also less susceptible to the infection caused by two of the variants currently causing the greatest concern in Britain, represented by the VOC 202012/01, with 109,093 confirmed and probable cases between 1 October 2020 and 1 March 2021, (case fatality 2.6% among the general population), and a South-African variant, VOC 202012/02 (also called B.1.351), accounting for 226 confirmed and 76 probable cases between 1 October 2020 and March 2021 (case fatality 2.3% among the general population) [108].

A study by Brookman et al. has focused on two groups of patients aged 18 or younger, admitted to King’s College Hospital during the two pandemic waves: twenty of them were admitted between 1 March 2020 and 31 May 2020, sixty between 1 November 2020 and 19 January 2021. The frequency of severe forms requiring ventilation support or oxygen therapy appears rare during both of these periods. The study has proved that, even though a smaller number of children and young adults was admitted to hospitals during the second wave of the pandemic, they do not have to suffer from a more severe infection form. This suggests that the infection caused by the B.1.1.7 variant seems no different from the one caused by the original strain [109].

On the other hand, paediatricians from Israel have reported that, contrarily to what happened during the first pandemic wave, “more than 50,000 children” were tested positive only in January. This might be related to the emerging of the more contagious UK variant: the Director of the Immunotherapy Laboratory in Israel has concluded that, since the daily number of cases among children younger than 10 has risen by 23% after the new variant appearance in Israel, even though it has not yet been shown that the latter is more dangerous for children than the original strain, it might be advisable to reopen schools gradually in order to follow the infection pattern more carefully [102].

Similar concerns have been raised in Corzano, a village in the province of Brescia (with a population of about 1500 individuals), where, on 3rd February 2021, 10% of the inhabitants were tested positive, with children from primary or nursery school accounting for 60% of the total [13]. Discussion about the new variant called B.1.617 (often called—Indian variant) is not the aim of this review, but currently new updates and new scenarios about its transmission are emerging. According the ‘Our World In Data’, COVID-19 vaccination coverage in India, up to the 10 May 2021, is 9.7% of the total population with at least one dose. In European countries, instead, the median one dose vaccination uptake is 31.6% of the total population [110].

Such a low vaccination coverage is probably the main reason for the increased mutation rate that occurred in SARS-CoV-2. In India, evolution and adaptation related SARS-CoV-2 diversification has been recently observed. There are three distinct Indian lineages within B.1.617, with distinct mutation profiles: B.1.617.1, B.1.617.2 and B.1.617.3 [111,112].

Lineages B.1.617.1 and B.1.617.2 were reported in India since December 2020; over the past eight weeks, there was an increase in the number of reported cases and deaths. According to the literature, there is very little information about the severity of B.1.617 lineage in the other countries [113]. No deaths, among those affected by any of B.1.617 lineages, have been reported in United Kingdom until 7 May 2021 [114]. Lineage B.1.617.3 has been firstly detected in India in February 2021; later, it was found in the United Kingdom, Russia and USA [115]. Even though all three lineages contain the mutation L452 R and D614G, both associated with increased transmissibility, lineage B.1.617.2 seems to be as transmissible as B.1.1.7 (the dominant UK variant) [116].

Further studies are still underway, and available data are not sufficient to assume that there may be an increased risk of viral susceptibility and spread among children and young people in general. Again, the disease caused by variants of the virus, in children, seems to have the same characteristics as the original strain.

## 9. “What Doesn’t Kill Me Makes Me Stronger”: Are We Sure? Psychological Aspects of the Pandemic

On 16 June 2020, the Italian Ministry of Health informed that the lockdown phase ‘which had just been completed’ led to a situation of stress among children and teenagers in our country, with negative consequences not just in terms of their physical health, but also on their emotional–psychological condition [116]. The Undersecretary of State for Health underlined that these were the conclusions from an Italian survey, conducted at the Gaslini Hospital (Genoa), on the psychological impact of the COVID-19 pandemic in Italian families [117]. The study analysed data from online questionnaire involving 6800 respondents.

Relevant behavioural changes were reported in two groups of children, aged < 6 and 6–18 years, with a rate of 64.3% and 72.5%, respectively. The first group also showed increased irritability (34.7%), restlessness (18.6%) and separation anxiety (16.4%), while in the second group, the behavioural changes more frequently entailed somatic components, for example shortness of breath (71.3%) and difficulty in getting asleep then awake again. It also appeared (using the SleepScore, SubUse and COVIDstress methods) that the severity of dysfunctional behaviours in both groups was closely related to the distress experienced by the parents themselves in dealing with quarantine (with a significant *p*-value of <0.0001). Since June 2020, the Italian freephone helpline for psychological assistance received over 50,000 calls, and 9.5% out of the total came from students [116].

The survey by Esposito et al. [118] has shown a more severe psychological impact of COVID-19 among females (the male gender seems to be a protective factor against negative feelings) and among students living in regions where the incidence of COVID-19 is higher. More specifically, a feeling of sadness appeared to be remarkably more frequent among females (84%) than in males (68.2%), and especially significant in the 14–19 years age range; it has been assumed that the primary cause of this feeling might be loneliness, associated with the lack of social contacts generally established in a school setting. Finally, 48.7% of girls reported that they felt like crying every day (compared to 13.4% in males, *p*-value < 0.001). A study conducted in the region where “everything began”, Hubei, collected data from 1784 students after an average of 33.7 days in lockdown, reporting symptoms of depression and generalized anxiety in 22.6% and 18.9% of cases, respectively [119].

Saurabh et al. [120] interviewed 121 subjects, children and teenagers, average age 15.4 years, who said that their prevailing thoughts were associated with feelings of anxiety (68.6%), hopelessness (66.1%) and concern about the future (61.9%).

A recent study by Qin et al. [121] covered a large section of the population (almost two million subjects) in the province of Guangdong, whose average age was 12 years; through a questionnaire, the study aimed at assessing the mental health outcome between 8 March 2020 and 30 March 2020. A total of 10.5% subjects reported a significant psychological distress and, among them, 51.5% were girls. Moreover, as previously mentioned with regard to Italian students, the negative impact in terms of mental health appears to have been higher in regions with a higher positivity rate, compared to those with a lower incidence of the virus.

Before the COVID-19 pandemic, the prevalence of mental health disorders among children and adolescents was 13.4% [122]; now, one year after the global epidemic began, there are still few longitudinal studies, nevertheless the conclusion is that an increase in disorders related to generalized anxiety and episodes of depression has been reported and will continue to be recorded in the near future [123].

With regard to parents’ “thoughts” regarding this new “distance learning” approach and its impact on their children, a study [124] involving 6.720 parents from 7 different countries showed that 19.2% considered the quality of online teaching to be extremely poor, and 45% perceived the support provided by schools to students learning from home as lacking.

Furthermore, parental welfare was part of the assessment by Cusinato et al. [125], who concluded that many mothers perceived low levels of well-being and self-control and exhibited higher levels of anxiety compared to the general population. This family distress situation was also highlighted in a paper by Marchetti et al. [126], associating parent’s psychological distress as a risk factor for the development of externalizing problems and hyperactivity in children.

Finally, in a currently under review study [127] 69 patients were interviewed (43 adults and 26 children) who had participated in an earlier transversal neuro-imaging study regarding the development of social emotions. The scores for depression reached clinically significant levels in 32.56% of respondents (4.65% of them meeting at least one of the criteria for diagnosing a major depression episode).

## 10. Conclusions

With this literature review and worldwide overview, we tried to understand whether keeping schools open during the SARS-CoV-2 pandemic might be a key element in the increase in the infection trend. Despite many sources of reflection, a univocal and certain answer to this question has not clearly emerged. Describing COVID-19 spread in the school setting is a complex undertaking, especially due to the heterogeneity in the studies available in the literature. In fact, they deeply differ and are scarcely comparable, especially regarding the number of involved subjects, the timing of the studies, the SARS-CoV-2 screening guidelines and swabs’ management. In fact, in some of the analysed studies, the number of enrolled subjects varies from hundreds to thousands, and this makes the results hardly comparable.

Moreover, regarding the timing of the studies, those we considered have been conducted in variable period during this year of pandemic, in which the global trend might certainly have had a substantial impact in determining the infection rate in the paediatric population and in the school setting. In addition, the duration of the observation time has also been wide ranging, with studies varying in length from one to three months. Finally, in the case of more recent studies, we should consider two new variables which might change the current scenario, namely the new variants of the virus and the roll-out of the vaccination campaign.

The different guidelines in swab management, varying from one country to another, also constitutes a limitation. In most of the studies, to describe secondary transmission in schools, a screening of close contacts for the index-case was performed, with infection rates which sometimes greatly differed one from the other, paving the way for further considerations.

The large discrepancy in terms of incidence and secondary transmission among the various schools, depending on the country and social context, inevitably highlights the fragility of prevention systems (hand hygiene, physical distancing and wearing face masks), which were proven difficult to implement in very young paediatric subjects as well as among the whole school population; this might also be a reason underlying the encountered divergences.

In conclusion, the shortage of experimental studies covering interventions which might provide evidence of a closer association between SARS-CoV-2 infection and the variables under consideration, as well as the decisions taken by most governments worldwide with regard to the opening and closure of schools, based on the models of the A and B influenza virus, do not yet allow for specific answers to our aforementioned questions. Unlike influenza, where the virus might be a reservoir for the infection, SARSCo-V2 apparently spares children, and its spread in schools seems to be limited.

Moreover, primary school children have a more limited tendency to spread the virus among the population and regardless of the school cycle.

Data regarding the spread of COVID-19 worldwide seem to confirm that most of the children infected by SARS-CoV-2 present with mild forms of the disease, thus showing a markedly better prognosis compared to adults [128]. Nevertheless, some of the paediatric patients have developed a more serious form of the disease, which might require admission to the intensive care unit, although fatalities still constitute an exception.

The future of the pandemic in the paediatric population will of course take time to unfold; it will thus be necessary to regularly assess its epidemiology impact in relation to the changes in restrictive measures. Thus, although children do not seem to be the main vectors of SARS-CoV-2, it will still be necessary to keep our guard up in the near future, also taking into account the psychological effects of prolonged closures and the bias provided by the new unknown viral variants and the low predictable effects of vaccination.

## Figures and Tables

**Table 1:** Summary of the available studies investigating SARS-CoV-2 transmission in the school setting.

	Observation Time	Sample Size (n° of Subjects)	Incidence	Secondary Transmission	Detection Method	Main Findings	Ref.
Italy	14 September 2020–5 October 2020	65,104	1.8%	Not described	Nasopharyngeal swab	Low SARS-CoV-2 incidence in the first month of school reopening. 33.5% of cases in high school	[12]
	21 September 2020–4 December 2020	3431	0.5%	Not described	Nasopharyngeal swab	Long-term observations on two schools in Rome	[13]
	14 September 2020–15 October 2020	994	6.6% High Schools. 0.38% Elementary Schools	3.8%	Nasopharyngeal swab	Significant number of infections in the school setting	[14]
Sweden	24 February 2020–14 June 2020	1124	0.05%	Not described	Not described	Low number of infections by keeping schools open	[15]
Norway	20 August 2020–20 November 2020	234	0.9%	Not described	Not described	Schools with a low incidence of infections despite the second wave on-going	[16]
USA	31 August 2020–29 November 2020	5530	3.4%	3.7%	Not described	Intra-school transmission was reported less than general transmission in the country (Wisconsin)	[17]
	15 August 2020–23 October 2020	77,446	1%	4.1%	Nasopharyngeal swab	Eleven school districts in North Carolina participated with students in attendance. Findings: low incidence and secondary transmission	[18]
Australia	25 January 2020–9 April 2020	663	0.3%	1.2%	Nasopharyngeal swab	Low incidence and secondary transmission during the first SARS-CoV-2 wave	[19]
Germany	22 April 2020–15 May 2020	2037	0.6%	Not described	Sero-antibody test	Low susceptibility to infection in children	[20]
	25 May 2020–5 August 2020	3104	4.4%	0.5%	Nasopharyngeal swab	Through the use of masks and frequent ventilation of the buildings, school transmission remains low	[20]
Ireland	1 March 2020–13 March 2020	1001	no-described	0%	Nasopharyngeal swab	No cases were found among students and school staff	[21]
England	June 2020– July 2020	6727 Pupils 4628 Staff	11.2% 15.1%	Not described	Sero-antibody test	Infection rates reflect those of the general population. Therefore, children get infected in the same way of adults but are more asymptomatic	[22]
Israel	1 May 2020–14 June 2020	1161 Pupils 152 Staff	13.2%	Not described	Nasopharyngeal swab	Outbreak described ten days after school reopening	[23]
Chile	March 2020–April 2020	1009 Pupils 235 Staff	9.9% Pupils 16.6% Staff	Not described	Sero-antibody test	Described a school outbreak with high incidence in school staff	[24]

## Data Availability

Not applicable.
